# Effects of a brief psychodynamic intervention on depressive patients. The “unfreezing” of psychic activity

**DOI:** 10.1192/j.eurpsy.2022.759

**Published:** 2022-09-01

**Authors:** H. Haliday, M. Reynaud, B. Lignier

**Affiliations:** 1 UNIVERSITY OF BURGUNDY, Psychology, DIJON, France; 2 Espace psychothérapique du CHS La Chartreuse, Psychology, DIJON, France

**Keywords:** Psychotherapy, group, psychodynamic, Depression

## Abstract

**Introduction:**

While psychotherapy is an essential aspect of the treatment of depression, there are few studies focusing on the effectiveness of psychoanalytic and psychodynamic group therapies for depressed patients.

**Objectives:**

In this presentation, we will study the effects of a brief, 4-session psychodynamic intervention (BPI) led by a group of therapists, as inspired by the Lausanne model.

**Methods:**

The patients were recruited in a therapeutic setting. A free consent form was completed and the ethics of research explained to each participant. Our sample consisted of 32 patients (average age = 43.81 years, sex ratio: 1M/ 4F). The therapists gathered data by completing several assessment scales after each therapy session: MADRS, ESM, EFP, HAQ-IT, EDICODE, Counter-Transfer Scale. The SPPS software (V21) was used to analyze the data.

**Results:**

The patients’ mean MADRS score dropped by more than 11 after the four sessions. This improvement matches a more positive and committed self-reported counter-transference of the therapists towards the patients. As their insight increases, patients show greater behavioral and psychic activity. We name this exit of the depressive inhibition the “unfreezing” process. It enables more satisfactory human interactions and a more focused and structured self-narrative.

**Conclusions:**

BPI led by a group of therapists seem to be an effective therapeutic adjuvant in the “unfreezing” of the psychic processes in depressive patients. Our results point out the importance of jointly aiming at symptomatic improvement and therapeutic alliance.

**Disclosure:**

No significant relationships.

